# AGE DIFFERENCES IN EVERYDAY DISCRIMINATION AND CORTISOL DYNAMIC RANGE IN DAILY LIFE

**DOI:** 10.1093/geroni/igad104.1664

**Published:** 2023-12-21

**Authors:** Lydia Ong, Patrick Klaiber, Jin Wen, Nicole Stuart, Nancy Sin

**Affiliations:** University of British Columbia, Vancouver, British Columbia, Canada; Tilburg University, Tilburg, Noord-Brabant, Netherlands; University of British Columbia, Vancouver, British Columbia, Canada; University of British Columbia, Vancouver, British Columbia, Canada; University of British Columbia, Vancouver, British Columbia, Canada

## Abstract

Extensive work has linked everyday discrimination with poorer health. However, research testing age differences in this relationship is limited. The current study examined everyday discrimination and cortisol dynamic range, and whether the association differed by age. Cortisol dynamic range is believed to reflect physiological responsiveness to external demands such as stressors, with a compressed range indicating less responsiveness. Participants were 250 community-based adults living in British Columbia (ages 25-88, mean=46 years, 64% White, 68% women). At baseline, participants completed the Everyday Discrimination Scale, followed by four days of at-home saliva collection 3x per day: upon waking, 30 minutes post-waking, and before bed. Cortisol dynamic range was calculated as the log-cortisol peak minus log-cortisol nadir across the four days. Multiple regression controlled for race/ethnicity, education, gender, medications, average length of waking day, and depressive symptoms. Results showed a negative association between age and cortisol dynamic range, such that older age was associated with a smaller range (b=-0.01, SE=0.003, p<0.001). There was no main effect of everyday discrimination on cortisol dynamic range. However, there was a significant interaction between age and everyday discrimination. Among younger adults, more frequent everyday discrimination was associated with a smaller cortisol dynamic range. In contrast, among middle-aged adults, more frequent everyday discrimination was associated with a larger range (b=0.37, SE=0.08, p<0.001). There was no significant association between discrimination and cortisol dynamic range among older adults. Findings indicate differing patterns in discrimination and cortisol dynamic range between younger, middle-aged, and older adults.

